# Dementia, Mild Cognitive Impairment and Ambulatory Care Utilization

**DOI:** 10.1002/alz.087548

**Published:** 2025-01-09

**Authors:** Yi Chen, Bryan D James, Ana W. Capuano, Mousumi Banerjee, Mellanie Springer, Julie Bynum, Francine Grodstein

**Affiliations:** ^1^ Rush Alzheimer’s Disease Center, Chicago, IL USA; ^2^ Department of Internal Medicine Rush University Medical Center, Chicago, IL USA; ^3^ Rush University Medical Center, Chicago, IL USA; ^4^ University of Michigan, Ann Arbor, MI USA; ^5^ University of Michigan Medical School, Ann Arbor, MI USA

## Abstract

**Background:**

Dementia is underdiagnosed by the healthcare system, and MCI is rarely identified. Thus, while interventions to reduce cognitive decline are becoming increasingly available, it is not clear how appropriate individuals will be evaluated for treatment opportunities. A first step to improving diagnosis is understanding how older individuals with dementia or MCI interact with the healthcare system, especially ambulatory evaluation and management (E&M) visits, the backbone of healthcare.

**Method:**

We investigated 2,116 community‐dwelling participants in four cohorts at the Rush Alzheimer’s Disease Center, all with linked Medicare Fee‐for‐Service claims. Each year from 2011‐19, we classified participants as no cognitive impairment (NCI), MCI, or dementia, based on their annual cohort evaluation; we then compared number of ambulatory E&M visits in claims over the next year across groups, controlled for demographics and comorbidities, using repeated measures generalized Poisson mixed effect models. We also considered annual likelihood of visiting different provider specialties, using logistic mixed effect models.

**Result:**

We included 8,672 person‐years (PY) of followup: 76% with NCI, 17% with MCI, and 7% with dementia. Average age over followup was 82 years, and 24% of PY was among Black participants; mean number of ambulatory E&M visits was 9.7/year (SD 6.9). Dementia was strongly associated with fewer E&M visits (23% fewer visits/year, p<0.001) but changes began even in MCI, which was associated with about 5% fewer visits (p = 0.02), compared to NCI. Participants with dementia and MCI were more likely to visit a dementia‐related specialist (odds ratio [OR] = 1.9, p<0.0001 for MCI; OR = 1.8, p = 0.009 for dementia) than those with NCI, although only a small minority (<30%) saw these specialists. Further, both dementia (OR = 0.2, p<0.0001) and MCI (OR = 0.6, p = 0.0001) were related to substantially lower likelihood of seeing any other medical specialist, indicating that even those with MCI were not receiving the full spectrum of healthcare.

**Conclusion:**

Impaired cognition was associated with fewer ambulatory E&M visits, and less complexity in healthcare; while these results were strongest for dementia, alterations appeared even with MCI. Finding ways to improve ambulatory care utilization for those with cognitive impairment is critical to identifying and supporting individuals who can benefit from treatment interventions.

